# Epithelial–mesenchymal transition, proliferation, and angiogenesis in locally advanced cervical cancer treated with chemoradiotherapy

**DOI:** 10.1002/cam4.751

**Published:** 2016-05-27

**Authors:** Leonardo Rojas‐Puentes, Andrés F. Cardona, Hernán Carranza, Carlos Vargas, Luis F. Jaramillo, Delma Zea, Lucely Cetina, Beatriz Wills, Erika Ruiz‐Garcia, Oscar Arrieta

**Affiliations:** ^1^ Clinical Oncology Group, Centro Javeriano de OncologíaHospital Universitario San Ignacio (HUSI)BogotáColombia; ^2^ Clinical and Translational Oncology GroupInstitute of OncologyClínica del CountryBogotáColombia; ^3^ Foundation for Clinical and Applied Cancer Research (FICMAC)BogotáColombia; ^4^ Department of PathologyHospital Universitario San Ignacio (HUSI)BogotáColombia; ^5^ Radiotherapy GroupCentro Javeriano de OncologíaHospital Universitario San Ignacio (HUSI)BogotáColombia; ^6^ Department of Medical OncologyInstituto Nacional de Cancerología de México (INCAN)México CityMéxico

**Keywords:** Angiogenesis epidermal growth factor receptor, cervical neoplasms, epithelial–mesenchymal transition, gene expression

## Abstract

We evaluated the association between epithelial–mesenchymal transition (EMT)‐derived markers and expression of proteins associated with cell proliferation and tumor growth, as well as their prognostic roles, in 61 patients (mean age 52 ± 10 years) with locally advanced cervical cancer, all of whom were treated with chemoradiation and intracavitary brachytherapy. We used immunohistochemical analysis to assess the expression of proteins targeted in our investigation. Various statistical analyses were then conducted to assess protein marker associations with survival outcomes. Forty‐six percent of the patients were positive for human papilloma virus. Median progression‐free survival (PFS) was 6.6 months (95% confidence interval [CI]: 4.0–9.1, whereas overall survival (OS) was 30.0 months (95% CI: 11–48). Multivariate analysis demonstrated that vascular endothelial growth factor (VEGF) (*P* = 0.002), epidermal growth factor receptor (EGFR) (*P* = 0.001), and TWIST2 (*P* = 0.001) expression levels, as well as a tumor size <6 cm (*P* = 0.02), influenced OS. Changes in TWIST2 levels and loss of E‐cadherin expression were correlated with VEGF and EGFR levels; furthermore, patients with high TWIST2 expression had shorter OS (*P* = 0.0001), as those with loss of E‐cadherin (*P* = 0.02). OS was even shorter when positive EGFR or VEGF expression was related with EMT markers (positive EGFR + negative E‐cadherin: median 14 months, 95% CI: 3–24; negative EGFR + positive E‐cadherin: median 31 months, 95% CI: 14–NA;* P* = 0.02.). The presence of EMT markers was associated with proliferative and pro‐angiogenic protein expression and influenced the prognosis of locally advanced cervical cancer.

## Introduction

Cervical cancer (CC) is the second leading global neoplasm in incidence and mortality. Annually, this disease causes approximately 234,000 deaths, 40,000 of which occur in developing countries 1. Inconsistencies in the incidence/mortality ratio between countries with different economic resources are a consequence of marginal investment in screening programs and limited access to vaccines against human papilloma virus (HPV), which is associated with 90% of invasive neoplasms 2. Although information regarding the epidemiology of the disease in Latin America is limited, CC is known to be the second most common cause of cancer‐related death among women from Colombia and Mexico 3, 4, where locally advanced tumors (clinical stages IB2 to IVA according to the International Federation of Gynecology and Obstetrics [FIGO] staging classification) account for 60% of cases; lesions in early stages generally predominate in countries with greater wealth 3, 4, 5. Since the 1990s, chemoradiotherapy (CRT) has been implemented as the standard therapy for patients with locally advanced CC, and the 5‐year overall survival (OS) has increased by 12% 6. Despite the positive impact of this intervention, approximately 35% of patients progress, and it is thus imperative to identify factors related to the biological behavior of the disease 6.

Approximately, 90% of cancers exhibit some degree of epithelial–mesenchymal transition (EMT) during their progression. Epithelial cells interact through cell–cell and cell‐extracellular matrix adhesion complexes, which involve cadherins and integrins, respectively. These molecules are attached to the actin and cytokeratin filaments, which form the rigid cytoskeleton. In the intact epithelium, cells exhibit a rigid structure and are immobile, whereas mesenchymal cells present a flexible organization with a tendency toward migration 7. Normal epithelial cells directly communicate with the stroma through organized filaments, and with adjacent cells through *tight junctions* and *gap junctions*. The function of these connections is to restrict proliferation, modulate migration, and maintain cell polarity. Epithelial cells are in contact with bodily fluids in the apical surface of most tissues which contain growth factors which have receptors located within the basolateral surface. *Tight junctions*, on the other hand are composed of epithelial‐cadherin (E‐cadherin, encoded by *CDH1*) complexes that restrict the access of fluids into those spaces and stromal compartments 8, 9.

In neoplasms, including CC, the structural system undergoes a range of events that result in the transition of a polarized, cubic, and immobile epithelial cell into nonpolarized and unstable spiculated cells with the capacity for invasion and migration 7, 9. EMT is defined by the loss of epithelial markers, such as E‐cadherin, and low regulation of specific cytokeratins. Additionally, EMT is characterized by the overexpression of nominal mesenchymal elements, such as fibronectin, N‐cadherin, and vimentin, as well as the acquisition of an invasive fibroblastoid phenotype 8, 10.

EMT may be induced by intrinsic (somatic or germ‐line mutations and methylation) or extrinsic signals. Extrinsic signals include transforming growth factor beta, hepatocyte growth factor, epidermal growth factor, and fibroblast growth factor overexpression 11, 12, 13, 14. The loss of E‐cadherin is related to a higher risk of metastasis, increased tumor grade, and lower OS 15, 16, 17, 18.

HPV oncoprotein E6 mediates p53 inactivation, promotes vascular endothelial growth factor (VEGF) overexpression, and modulates angiogenesis by inhibiting thrombospondin 1 (TSP1), an adhesion glycoprotein that facilitates interactions with the stroma, allowing the binding of molecules such as fibrinogen, fibronectin, laminin, collagen type 5, and integrins *α*5 and *β*1, all of which are important in CC 19. Several studies have proved that the deregulation of these processes negatively affects the prognosis of CC patients, as well as the responses to certain treatments, including radiotherapy 19, 20. Noordhuis and collaborators demonstrated that increased epidermal growth factor receptor (EGFR) in women with locally advanced tumors treated with CRT was negatively associated with progression‐free survival (PFS). Moreover, altered EGFR immunoreactivity, including the phosphorylated fraction, was useful in predicting the response to treatment independently of tumor stage and histological pattern 21.

Establishing the status of key markers associated with angiogenesis and cellular proliferation, as well as their relationship with EMT in locally advanced CC treated with CRT, may enable the identification of high‐risk subgroups and the development of models to characterize and predict disease evolution. In this report, we describe the relationship between VEGFR and EGFR expression and key EMT markers in a population of women with CC that was treated with CRT and brachytherapy, establishing the impact over a range of outcomes.

## Materials and Methods

Patient information was obtained from clinical records of 61 women with CC at stages IB2–IVA who were treated homogeneously in Colombia. Several clinical variables (Table 1) were considered. Multiple outcomes were also assessed, including vital status at the end of the follow‐up, overall response (OR), PFS, OS, and mortality (based on RECIST 1.1 criteria) 22. Consent was obtained from all participants. This study was approved by scientific and ethics committee of Hospital Universitario San Ignacio and Instituto Nacional de Cancerología de México (N of approval: CEI/844 and CEI/844/13, respectively).

**Table 1:** Demographic, clinical, and pathologic characteristics of patient population

Variable	*N* (%)
Age (mean, SD)	52 (±10)
Stratified age	
<65 years old	56 (91.8)
>65 years old	5 (8.2)
Functional condition (KI)	
<70	10 (16.4)
>70	37 (60.7)
NA	14 (23.0)
Education grade	
Primary	14 (23.0)
High School	9 (14.8)
Vocational/Technical	13 (21.3)
Postgraduate	1 (1.6)
None	10 (16.4)
NA	14 (23.0)
Smoking history	
Non‐smoker	29 (47.5)
Smoker	12 (19.7)
NA	10 (16.4)
Number of years exposed to smoke (mean, SD)	12 (±7)
HIV (+)	0
Preneoplastic lesion history	
Yes	13 (21.3)
No	19 (31.1)
NA	29 (47.5)
HPV infection history	
Yes	28 (45.9)
No	7 (11.5)
NA	26 (42.5)
Histology	
Squamous	58 (95.1)
Adenocarcinoma	2 (3.3)
Adenosquamous	1 (1.6)
Histological grade	
Gx	18 (29.5)
G1	10(16.4)
G2	29 (47.5)
G3	4 (6.6)
Tumor size (mean, SD)	6.4 cm (±1.8)
Clinical condition (FIGO)	
IIA	2 (2.3)
IIB	29 (47.5)
IIIA	3 (4.9)
IIIB	23 (37.7)
IVA	2 (3.3)
Unknown	2 (3.3)
Positive pelvic ganglia (scissional biopsy or FNAB)	
Yes	5 (8.2)
Not evaluated	56 (91.8)

FNAB, fine needle aspiration biopsy; FIGO, international federation of gynecology and obstetrics; HIV, human immunodeficiency virus; HPV, human papilloma virus; KI, Karnofsky index; NA, not available; SD, standard deviation.

### Immunohistochemistry

Pathological evaluations were performed by two independent pathologists. Immunohistochemistry was carried out in tumor tissue implemented in the Translational Oncology Laboratory of the Foundation for Clinical and Applied Cancer Research (FICMAC, Bogotá, Colombia).

### Determination of VEGF expression

Paraffin sections of width 5 mm were sliced, placed onto an APES (3‐aminopropyltriethoxysilane)‐coated microscope slide, and deparaffinized. Samples were then treated with 0.3% hydrogen peroxide in methanol to block endogenous peroxidase activity. Antigen was subsequently recovered by immersing samples in a 10 mmol/L citric acid monohydrate buffer (pH 6.0) 0.01% (v/v). Samples were then cooled for 15 min, incubated for 30 min with normal rabbit serum (NRS, X‐0902 DAKO Inc., Carpinteria, CA), diluted 1.5 times in phosphate‐buffered saline (PBS) to reduce unspecific staining, and incubated for 60 min with polyclonal anti‐human VEGF (AB‐293‐NA, R&D Systems AG, Oxford, UK) diluted 1:100 in PBS. After three washes with PBS, samples were incubated for 30 min with biotinylated anti‐goat/‐rabbit antibodies (RAG, E‐0466, DAKO Inc., Carpinteria, CA) diluted 1:350. Samples were then exposed to streptavidin‐biotinylated horseradish peroxidase complex (DAKO GmbH, Glostrup, Denmark) for 30 min, and diaminobenzidine tetrachloride (SIGMA AG, Poole, Dorset, UK) was used as a chromogen. Counterstaining was carried out using Mayer's hematoxylin. A known positive sample was used as a positive control, and the primary antibody was excluded for a negative control. To assess VEGF expression, the methods used by Kimura and collaborators 23 were followed. Briefly, using a 200× magnification (0.12 mm^2^) camera, VEGF expression was semiquantitatively graded in two groups: <10% and ≥10% of stained tumor cells, deemed negative and positive expression, respectively.

### Determination of EGFR expression

Samples were processed as described previously for paraffin treatment. EGFR staining was performed using 1:100 PBS‐diluted polyclonal rabbit IgG against the receptor (Santa Cruz Biotechnology Inc., Santa Cruz, CA). Breast carcinoma samples were used as positive controls. Negative controls consisted of the same cervical tissue samples not exposed to the primary antibody. Samples were considered positive when staining was at least twice as high on a scale of 1–4 +  and if at least 10% or more of the tumor cells were stained relative to healthy tissue or the negative control 21.

### Determination of TWIST2

The paraffin‐embedded tissue was exposed to mouse monoclonal anti‐TWIST2 (3E10, ABCAM, UK). Samples were analyzed to establish cytoplasmic immunoreactivity measured by intensity on an arbitrary scale from 0 to 3 (0 negative; 1 weak; 2 moderate; and 3 strong) 24.

### Determination of E‐cadherin

The tissue was exposed to monoclonal rabbit anti‐E‐cadherin antibody (EP700Y, ABCAM, UK), and cellular membrane reactivity was measured. Immunoreactivity was classified as follows: 0, negative (<10% positive stained cells); 1, low reactivity (70% positive cells); 2, moderate reactivity (>70% low‐medium stained cells); and 3, strong reactivity (>70% high stained cells) 24.

### Statistical analysis

Results were obtained by determining absolute and relative frequencies, central tendency measures, and dispersion. Bivariate analysis was performed with contingency tables subject to dependence and association tests by using Cochran's Q paired analysis in three groups and two categories, with significance set at *P* < 0.05. Survival estimates were implemented using the Kaplan–Meier nonparametric model and compared using the log‐rank and Breslow tests. To assess survival‐influencing factors, a Cox (stepwise model) multivariate analysis was performed. This model was adjusted for clinical stage, histology, age, history of previous HPV infection, smoking history, and tumor size.

## Results

### General characteristics of the population

The study population included 61 women aged between 29 and 75 years old; most were in a good functional state. Half of the patients had no tobacco‐smoke exposure history; the mean smoking period among the 12 women who did smoke was 12 years. Regarding history of preneoplastic lesions, it was not possible to obtain data from previous screening studies in 47% of cases, and positive HPV was documented for 28 patients. No HIV infection was recorded. The most frequent histological type was squamous carcinoma (*n* = 58), which was moderately and poorly differentiated in 47.5% and 6.6% of cases, respectively. A total of 51% (*n* = 31) of cases exhibited a tumor volume >6 cm, and according to the FIGO classification, 2.3%, 47.5%, 4.9%, 37.7%, and 3.3% of patients were at stages IIA, IIB, IIIA, IIIB, and IVA, respectively, at the time of diagnosis. Only five women had pelvic ganglia invasion. Table 1 summarizes the main characteristics of the cohort.

Teletherapy was suspended in 25 patients due to toxicity (diarrhea, 12 cases; actinic cystitis, 4 cases) and in 12 women due to limitations in healthcare access. A total of 75% of the subjects completed high‐dose brachytherapy, and the average administration of cisplatin cycles during concomitance was 4 ± 1 (standard deviation). After oncologic treatment, 47% achieved complete clinical and imaging response and 25%, a partial response (the OR was 67%). Eight patients exhibited stable or progressive disease, whereas information regarding final treatment outcome was impossible in nine patients. The median monitoring period was 13.2 months (95% confidence interval [CI]: 5.9–99.6). During this period, disease progression was documented in 31 cases. Data S1 shows the outcomes after treatment.

### Overall and progression‐free survival

Median OS and PFS were 30.2 months (95% CI: 11.6–48.8) and 6.6 months (95% CI: 4.0–9.1), respectively (Data S2A and S2B). When sorted by tumor size, there were no significant differences in PFS (8.7 and 9.1 months in lesions <6 and ≥6 cm, respectively; *P* = 0.85), while there were significant differences in OS (71 and 24 months in lesions <6 and ≥6 cm, respectively; *P* = 0.02) (Supplementary 2C). Patients who achieved complete imaging response showed significantly higher PFS (*P* = 0.0001) and OS (*P* = 0.0001). There was no significance when PFS was sorted according to tumor grade (*P* = 0.23), although there was a significant difference according to the FIGO classification, where PFS was longer without evidence of progression among patients in stages IIA and B; *P* = 0.004). OS showed similar results but was also influenced by tumor grade (*P* = 0.01). The cause of death in 33 cases was related to disease progression, especially at the locoregional level. In two women, the cause of death was not determined. Multivariate analysis did not shown any influence of clinical variables on PFS or OS.

### Relationship between EMT, cellular proliferation, and angiogenesis‐associated gene expression

A total of 46% of patients exhibited EGFR immunoreactivity independent of tumor size, disease grade, and histological grade (*P* = 0.49, *P* = 0.40, and *P* = 0.12, respectively). Among EGFR‐positive cases, 57% showed VEGF reactivity; this correlated negatively with OS (*P* = 0.0001) in this population (*n* = 16).Immunoreactivity (strong and moderate expression) to TWIST2 was observed in 59% of women (*n* = 36); 34.4% of cases (*n* = 21) were associated with EGFR positivity and 31% (*n* = 19) with VEGF increase. Additionally, 18% of the patients (*n* = 11) exhibited a loss of E‐cadherin, whereas 9.8% (*n* = 6) had positive EGFR expression. Only 6.6% of cases (*n* = 4) showed E‐cadherin loss and higher VEGF expression. Increased TWIST2, EGFR, and VEGF expression was found in 11.5% of samples, while E‐cadherin loss with increased EGFR and VEGF was observed in 4.9%. Tables 2 and 3 show the expression of EMT markers and their association with the main cellular proliferation and angiogenic markers.

**Table 2:** Protein expression of epithelial–mesenchymal transition markers

TWIST2 expression		E‐Cadherin expression	
Value	*N* (%)	Value	*N* (%)
Negative	14 (23)	Positive	13 (21.3)
Weak	8 (13.1)	Low‐moderate	34 (55.7)
Moderate	17 (27.9)	Absent	11 (18.0)
Strong	19 (31.1)	No data	3 (5.0)
No data	3 (4.9)		
Total	61 (100)		61 (100)

**Table 3:** Epithelial–mesenchymal transition‐related protein expression associated with EGFR/VEGF

EGFR (+)/TWIST2 (+)		VEGF (+)/TWIST2 (+)
** **	*N* (%)	*N* (%)
Yes	21 (34.4)	19 (31.1)
No	34 (55.7)	40 (65.6)
No data	6 (9.8)	2 (3.3)

EGFR (+)/E‐Cadherin (‐)		VEGF (+)/E‐Cadherin (‐)
** **	N (%)	N (%)
Yes	6 (9.8)	4 (6.6)
No	48 (78.7)	52 (85.2)
No data	7 (11.5)	5 (8.2)

EGFR (+)/VEGF (+)/TWIST2 (+)		EGFR (+)/VEGF (+)/E‐Cadherin (‐)
Yes	7 (11.5)	3 (4.9)
No	53 (86.9)	57 (93.4)
No data	1 (1.6)	1 (1.6)

EGFR, epidermal growth factor receptor; VEGF, vascular endothelial growth factor.

### Clinical outcomes of interest according to the expression of EMT markers and cellular proliferation and angiogenesis genes

**Figure 1:**
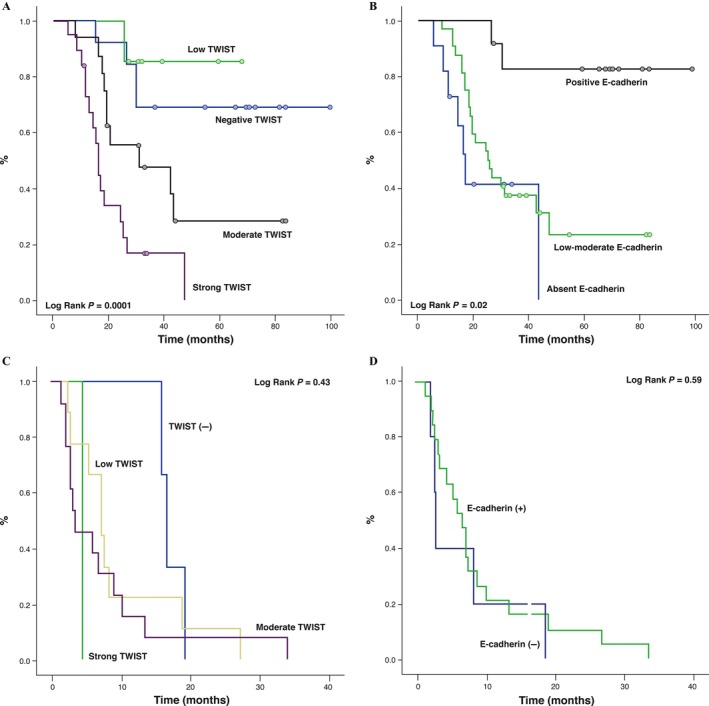
Survival curves according to epithelial–mesenchymal transition markers. (A) Overall survival by TWIST2 expression. (B) Overall survival by E‐cadherin expression. (C) Progression‐free survival by TWIST2 expression. (D) Progression‐free survival by E‐cadherin expression.

OS was associated with the presence of EMT markers (TWIST2 and E‐cadherin), specifically, TWIST2 positivity had a negative effect on OS. Patients with increased reactivity showed an OS of 16 months (95% CI: 14.0–18.0), compared with 76 months (95% CI: 58.0–95.0) in TWIST2‐negative women (*P* = 0.0001) (Fig. 1A). Similarly, OS was higher in the E‐cadherin‐expressing population (87 months [95% CI: 71.0–102.0] vs. 17 months [95% CI: 13.0–21.0]; *P* = 0.02) (Fig. 1B). PFS was not affected by any EMT markers (Figs. 1C and D).

**Figure 2:**
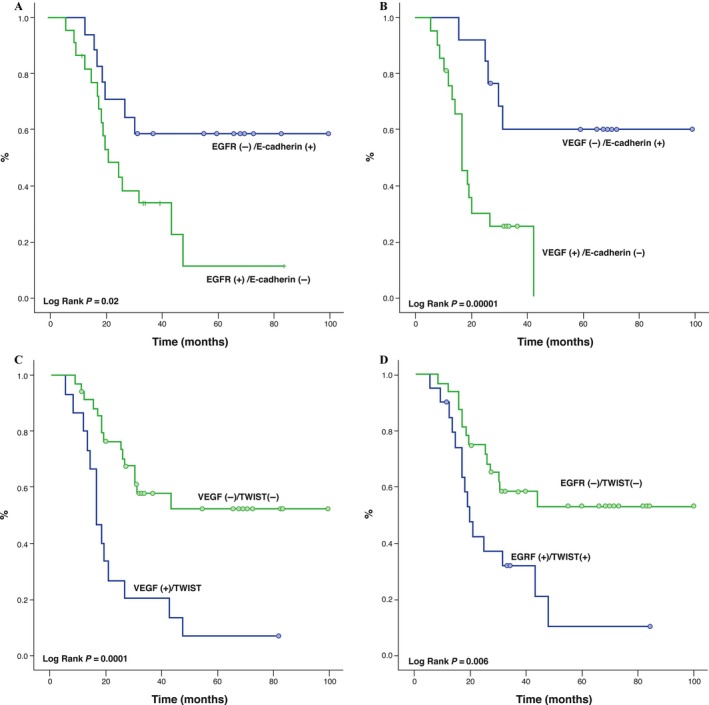
Survival curves for epithelial–mesenchymal transition markers and tumor markers. **(**A) Overall survival by epidermal growth factor receptor (EGFR)/E‐cadherin expression. (B) Overall survival by vascular endothelial growth factor (VEGF)/E‐cadherin expression. (C) Overall survival by EGFR/TWIST2 expression. (D) Overall survival by VEGF/TWIST2 expression.

The positive association of proliferation and angiogenesis (EGFR and VEGF) with EMT markers had a higher impact on OS than each of the biomarkers individually. Observed OS was 14 months (95% CI: 3.0–24.0) with the combination EGFR (+)/E‐Cadherin (‐), compared to 31 months (95% CI: 14.0) with EGFR (‐)/E‐Cadherin (+) (*P* = 0.02) (Fig. 2A). Similarly, VEGF (+)/E‐cadherin (‐) had a negative effect on OS (14 months, 95% CI: 3.0–25.0) compared to VEGF (‐)/E‐Cadherin (+) (42 months, 95% CI: 25; *P* = 0.00001) (Fig. 2B).

**Figure 3:**
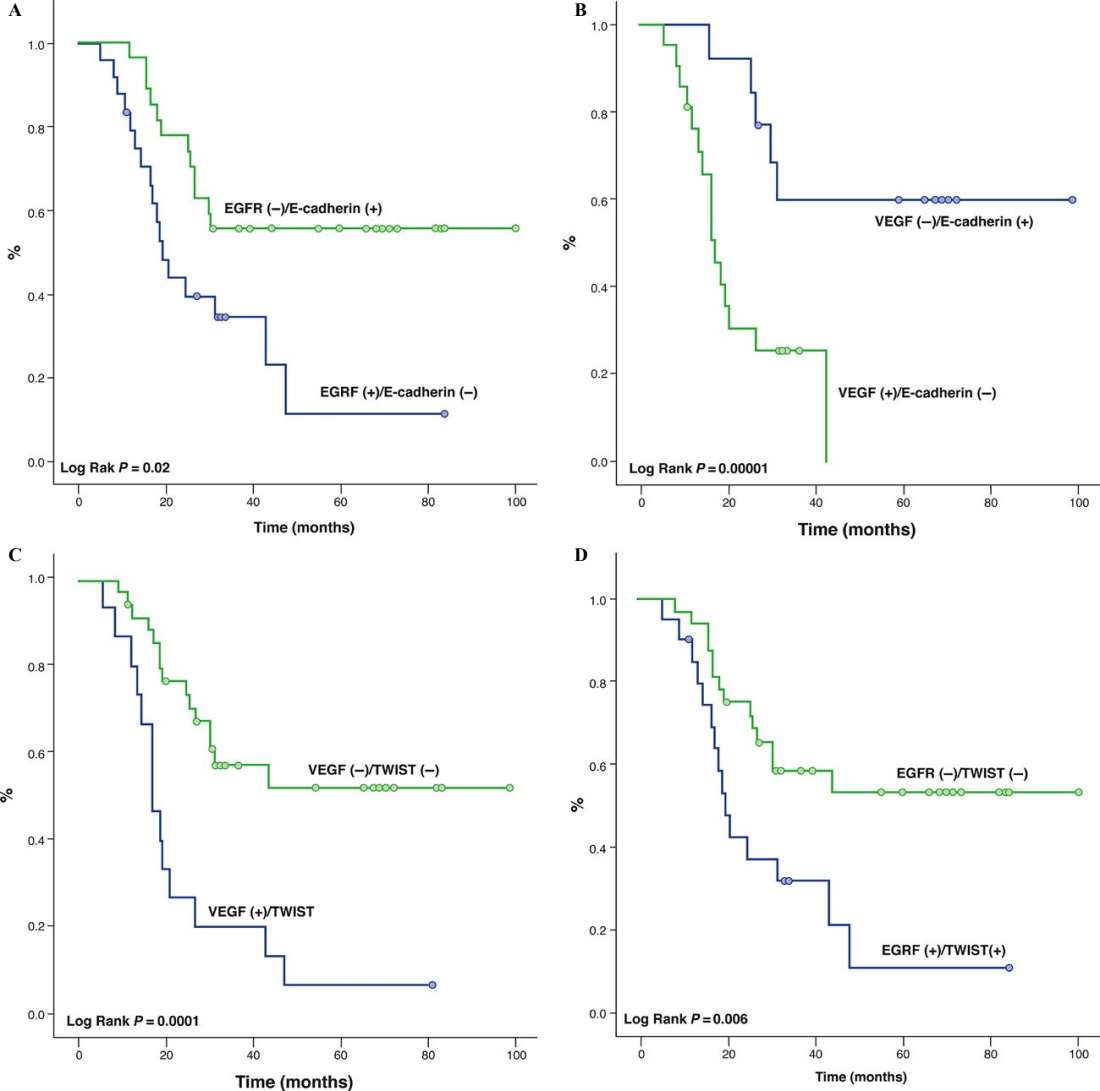
Survival curves for epithelial–mesenchymal transition markers and tumor markers. (A) Overall survival by epidermal growth factor receptor (EGFR)/E‐cadherin expression. (B) Overall survival by vascular endothelial growth factor (VEGF)/E‐cadherin expression. (C) Overall survival by EGFR/TWIST2 expression. (D) Overall survival by VEGF/TWIST2 expression.

Coexpression of TWIST2 and EGFR or VEGF had similar effect on OS as previously described for E‐cadherin (Figs. 3C and D). Coexpression of all three markers (TWIST2, EGFR, and VEGF) also had a significant impact on OS (Data S3). At the end of the monitoring period, 57% of women were deceased, an outcome that was influenced by EGFR, VEGF, and TWIST2 expression in the multivariate analysis. E‐cadherin loss exhibited a marginal *P*‐value (Table 4).

**Table 4:** Overall survival multivariate analysis (Cox model)

Variable	*P*	95% CI	
		Lower	Upper
TWIST2	0.001	1.621	6.747
E‐Cadherin	0.052	1.035	2.173
EGFR+/VEGF+/TWIST+	0.663	0.191	2.863
EGFR	0.015	1.661	12.660
Microvascular density	0.704	0.153	3.556
HIF‐1*α*	0.15	0.52	54.5
VEGF	0.002	1.419	4.684
Tumor size	0.073	0.928	5.245
Complete imaging response	0.40	0.97	1.00
FIGO	0.525	0.470	1.470
HPV infection	0.244	0.350	1.306
Basal ECOG	0.868	0.328	3.755
Ganglion compromise	0.126	0.198	1.221
Tobacco exposure	0.873	0.416	2.809

CI, confidence interval; ECOG, Eastern cooperative oncology group; EGFR, epidermal growth factor receptor; FIGO, international federation of gynecology and obstetrics; HIF‐1*α*, hypoxia‐inducing factor‐1*α*; HPV, human papilloma virus; VEGF, vascular endothelial growth factor receptor.

## Discussion

CC represents one of the leading causes of incidence and mortality due to cancer among women in Latin America 3, 4, 5. Regular screening using cervicovaginal cytology has decreased its frequency in developed countries. A systematic review of 25 clinical experiments (*n* = 3452) demonstrated that CRT administration reduces the risk of death within 5 years by 19%, with an absolute OS benefit of 10–12% 6. In this study, we found a response rate similar to previous literature, since we documented a 3‐year OS of 66%, which is equivalent to earlier analysis of concomitant therapy in women with locally advanced CC.

CC results from the abnormal expression of several genes modified by the HPV E6 and E7 oncoproteins. The outcomes of and radiotherapy are predicted by differences in protein expression in cervix‐derived tumor cells. Changes in 52 conductor genes determine the biological behavior of squamous neoplasms, including EGFR, VEGF, hypoxia‐inducible factor (HIF), HYAL2, SV2A, and CASP10 25, 26. Furthermore, the overexpression of VEGF in radiotherapy‐treated IB2 and IVA tumors negatively influenced OS 27. Our study suggests that VEGF overexpression negatively affects survival, even after controlling for factors such as tumor size, histological grade, and local extension of pathology.

Research suggests that EGFR is altered in more than 80% of CC patients, and have been associated with poor prognosis due to its role in chemo and radiotherapy responses 27. Liang and colleagues demonstrated that teletherapy increased receptor bioavailability and outsourcing; thus, blocking the receptor could explain the increased response in CC patients specifically treated with EGFR‐targeted drugs 28. Several studies have described synergistic effects of cytostatic drugs when combined with erlotinib in different lineages, including multiresistant cervical cancer 518A2 cells 29, 30. Approximately, 46% of Colombian women with CC exhibit EGFR overexpression, and while this did not influence PFS in our study, it negatively affected OS. Increased EGFR expression was also associated with higher microvascular density (data not shown), and with significantly higher VEGF and HIF‐1*α* expression. On the other hand, there was no association between EGFR immunohistochemical score and tumor size.

EMT markers have been associated with greater tumor invasion and progression in women with CC 31. In particular, HPV E6 oncoprotein expression induces characteristic changes in EMT 32. Myong and collaborators showed that the loss of E‐cadherin and gain of vimentin are found within more aggressive cervical‐derived invasive lesions but not within preneoplastic alterations 33. Our results suggest that the presence of EMT markers negatively affected women with locally advanced CC. Specifically, TWIST2 and loss of E‐cadherin were associated with lower OS, suggesting the existence of a genotype‐specific cellular subpopulation that could benefit from the administration of molecular inhibitors.

Studies in cellular models have found an association between angiogenesis inductors (i.e., anoxia/hypoxia) and altered EMT proteins. For example, HIF‐1*α* induces EMT through modulators such as SNAIL1. Similarly, vimentin and TWIST1 expression have a positive feedback effect on HIF‐1*α* through the activation of growth receptors related to the PI3K/mTOR/AKT signaling pathways 34, 35. Studies of xenografts in preinvasive cells demonstrated that the addition of VEGF induces the appearance of EMT markers 36, 37. Our study showed that the increase in VEGF expression is proportional to that of EMT markers in vivo, thereby, shortening OS. The expression of EMT markers is important in CC patient candidates for anti‐VEGF treatment. Recently, Tewari and colleagues showed that the addition of bevacizumab to chemotherapy in recurrent or metastatic CC patients decreases the risk of death by 19% 38. Additionally, EMT induction may be the main route for acquired resistance to anti‐VEGF 39.

EGFR overexpression has been associated with worse prognosis in locally advanced and metastatic CC patients 21. Moreover, CC patients with increased EGFR expression exhibit lower PFS and OS 21. Our study was consistent with these findings as assessed independently by multivariate analysis. Moreover, hypoxia increases EGFR expression, which changes the cellular phenotype to a mesenchymal variety 22. We found that EGFR overexpression was associated with an increase in TWIST2 and a loss in E‐cadherin. The presence of these three markers (VEGF‐measured angiogenesis, EGFR overexpression, and positive EMT) had a significant negative impact on OS in our population. Studies have shown that EMT is one of the main resistance mechanisms to anti‐EGFR therapies 40. Our study indicates that patients with EGFR overexpression have EMT, which might preclude the use of anti‐EGFR drugs. Moreover, basal measurement of EMT features within tumor tissues obscures the phenotypic changes induced by CRT, especially in women who have disease stability or progression.

A main limitation of this study included sampling bias since all cases were obtained from a single institution, which may influence the lack of relationship between protein expression and PFS. Furthermore, the absence of pathological evaluation prior to and after intervention is another limitation, as is heterogeneity in gene expression. State‐of‐the‐art techniques that allow better quantification of biomarkers within paraffin‐embedded tumor tissues may be able to determine gene expression more robustly. Another limitation of our study is the amount of missing data regarding formal clinical evaluation and HPV testing of the cohort.

## Conclusions

Four conditions negatively affected OS without modifying PFS: the expression of EGFR, VEGF, and TWIST2. These results will help to determine the prognosis of locally advanced CC; hopefully, these factors might be regulated using antiangiogenesis and/or therapies inhibiting the EGFR pathway. EGFR/VEGF expression determines disease prognosis, particularly when E‐cadherin loss and TWIST2 overexpression are evident. These latter two variables modify OS individually or in conjunction with increased EGFR/VEGF expression. HPV infection may also induce EMT by encouraging EGFR‐dependent proliferation and angiogenesis.

## Conflict of Interest

Carlos Alberto Vargas was an external consultant at Laboratorio Productos Roche S.A, and Jorge Miguel Otero was the same at Glaxo Smith‐Kline. In the past, Andrés Felipe Cardona has received stipends from Pfizer S.A, Novartis de Colombia S.A., Boehringer Ingelheim S.A., and Productos Roche S.A. Carlos Vargas, Hernán Carranza, Jorge Miguel Otero, and Andrés Felipe Cardona are members of the Foundation for Clinical and Applied Cancer Research (Fundación para la Investigación Clínica y Molecular Aplicada del Cáncer – FICMAC). The Cervical Cancer Research Platform was designed and implemented, thanks to the full support from the Foundation for Clinical and Applied Cancer Research (FICMAC; stipend 011‐2009) and the grants Buen‐D and Silberman for Cancer Research. Leonardo Rojas‐Puentes received the Carlos Slim Promotion of Health Research 2012 grant (Carlos Slim para Impulso a la Investigación en Salud 2012), which supported the author for the work described in this manuscript.

## Supporting information


**Data S1.** Main outcomes after chemoradiotherapy and brachytherapy.
**Data S2.** Survival curves within the study population. (A) Overall survival. (B) Progression‐free survival. (C) Overall survival by tumor size.
**Data S3.** Overall survival by TWIST2, EGFR, and VEGF expression.
