# Supplementary material for: Epithelial–mesenchymal transition, proliferation, and angiogenesis in locally advanced cervical cancer treated with chemoradiotherapy
Source: Cancer Med. 2016 May 27;5(8):1989–99. doi: 10.1002/cam4.751 (PMC4884920; doi:10.1002/cam4.751)
Supplement: Supplementary file 1 — Data S1. Main outcomes after chemoradiotherapy and brachytherapy. Data S2. Survival curves within the study population. (A) Overall survival. (B) Progression‐free survival. (C) Overall survival by tumor size. Data S3. Overall survival by TWIST2, EGFR, and VEGF expression. [file CAM4-5-1989-s001.doc]

**Supplementary 1.** Main outcomes after chemoradiotherapy and brachytherapy.

| **Outcomes** | **N (%)** |
| --- | --- |
| Unplanned termination of teletherapy    No    Yes, due to toxicity  Yes, due to other causes    NA | 33 (54.1)  13 (21.3)  12 (19.7)  3 (4.9) |
| Complete treatment with high-rate brachytherapy    Yes    No    NA | 46 (75.4)  11 (18.0)  4 (6.6) |
| Number of weekly cycles of carboplatin or cisplatin    1    2    3    4    5    6    NA | 2 (3.3)  3 (4.9)  7 (11.5)  10 (16.4)  16 (26.2)  21 (34.4)  2 (3.3) |
| Post-treatment clinical condition    Tumor absence    Residual tumor    Unknown | 28 (45.9)  19 (31.1)  14 (23.0) |
| Post-treatment tomographical condition    Complete response    Partial response    Stable disease    Progressive disease    Unknown | 29 (47.5)  15 (24.6)  5 (8.2)  3 (4.9)  9 (14.8) |
| Disease progression    Yes    No    Unknown | 31 (50.8)  23 (37.7)  7 (11.5) |

NA: Not available.

**Supplementary 2 1.** Survival curves within the study population. **(A)** Overall survival. **(B)** Progression-free survival. **(C)** Overall survival by tumor size.

**A.**

**B.**

**C.**

**Supplementary 3.** Overall survival by TWIST2, EGFR, and VEGF expression.
